# The Prevalence of Fabry Disease Among Turkish Patients with Non-Obstructive Hypertrophic Cardiomyopathy: Insights from a Screening Study

**DOI:** 10.4274/balkanmedj.galenos.2019.2019.5.125

**Published:** 2019-10-28

**Authors:** Hasan Ali Barman, Barış İkitimur, Burçak Kılıçkıran Avcı, Eser Durmaz, Adem Atıcı, Serkan Aslan, Serdar Ceylaner, Hakan Karpuz

**Affiliations:** 1 Clinic of Cardiology, İstanbul Okmeydanı Training and Research Hospital, İstanbul, Turkey; 2 Department of Cardiology, İstanbul University Cerrahpaşa School of Medicine, İstanbul, Turkey; 3 Clinic of Cardiology, İstanbul Taksim Training and Research Hospital, İstanbul, Turkey; 4 Clinic of Cardiology, İstanbul Mehmet Akif Ersoy Thoracic and Cardiovascular Surgery Training and Research Hospital, İstanbul, Turkey; 5 Intergen Genetic Center, Ankara, Turkey

**Keywords:** Echocardiography, Fabry disease, hypertrophic cardiomyopathy

## Abstract

**Aims:**

Fabry disease is an X-linked lysosomal storage disorder due to a deficiency of the α-galactosidase A enzyme. Cardiac involvement is present in over 60% of adult cases of Fabry disease. Hypertrophic cardiomyopathy without left ventricular outflow tract obstruction is the most common phenotype. The aim of the study was to screen adult patients with hypertrophic cardiomyopathy without left ventricular outflow tract.

**Methods:**

A total of 80 patients between the ages of 18 and 65 years old, were referred to a tertiary center for trans-thoracic echocardiography for various clinical indications. They were investigated for the presence of idiopathic left ventricular hypertrophy without resting or dynamic left ventricular outflow tract obstruction. Plasma α-galactosidase A enzyme activity and α-galactosidase GLA gene mutations were investigated.

**Results:**

The mean age was 41.5±12.7 years and 66.25% of patients were males. The mean echocardiographic parameters were as follows: left ventricular ejection fraction 60.7±7.4%, interventricular septum thickness 18.2±4.4 mm, left ventricular posterior wall 13.5±2.1 mm, left ventricular end-diastolic diameter 47.4±6.2 mm, left ventricular end-systolic diameter 27.8±6.5 mm, and left ventricular mass index 171.05±48.5 g/m². Hemizygous mutations associated with Fabry disease were detected in two male patients (2.50% of the screened population): NM_000169.2:c.334C>T(p.Arg112Cys), NM_000169.2:c.902G>A(p.Arg301Gln).

**Conclusion:**

Fabry disease should be considered in the differential diagnosis in a highly selected patient population with unexplained left ventricular hypertrophy. The cardiologist may play an important role in the screening and diagnosis of the disease.

Hypertrophic cardiomyopathy (HCM) is defined by the presence of increased left ventricular (LV) wall thickness that is not solely explained by abnormal loading conditions (1). The most common metabolic disorder in adults with HCM is Fabry disease (FD), with a prevalence of around 0.5%-1% in patients with HCM older than 35-40 years old (2). FD is a rare X-linked recessive hereditary lysosomal storage disorder due to α-galactosidase A enzyme (AGE) deficiency caused by various mutations in the GLA gene resulting in abnormal glycosphingolipid metabolism (3). Disorders, which may affect patients with FD and cardiac involvement, include HCM, ischemic heart disease, valvular diseases, and arrhythmias (4). Cardiac involvement is present in over 60% of adult cases of FD. The most common phenotype of FD has concentric HCM without LV outflow tract (5).

Recent interest in the diagnosis of FD among patients with HCM stems from the fact that FD has evolved to become one of the few genetic storage diseases with a medical therapeutic option in adult patients, with the advent of enzyme replacement therapy (6). The reported figures related to the frequency of FD among patients with unexplained LV hypertrophy (LVH) in different countries have been variable (7,8,9,10,11,12,13). Screening studies have great importance to estimate the prevalence of FD, especially in patients with kidney and cardiac disease, which are considered high-risk populations for the presence of FD. To the best of our knowledge, there are no screening studies published about the frequency of FD with cardiac involvement and unexplained LVH in Turkish patients. The aim of this study was to screen adult patients with unexplained LVH without resting or dynamic LV outflow tract obstruction for the presence of mutations known to be associated with FD.

## MATERIALS AND METHODS

We studied 80 consecutive unrelated patients between January 2013 and September 2017 with non-obstructive HCM (without resting or dynamic LV outflow tract obstruction) diagnosed by trans-thoracic echocardiography according to the criteria of the European Society of Cardiology Guidelines. The diagnosis of HCM was based on echocardiographic demonstration of unexplained LVH, i.e., maximum LV wall thickness of 15 mm in at least one myocardial segment as suggested by European Society of Cardiology Guidelines on Diagnosis and Management of HCM (4). As required for the establishment of the diagnosis of HCM, the presence of LVH should be unexplained by abnormal loading conditions (1,4). Patients with arterial hypertension were excluded when hypertension was associated with mild LVH (LV mass/body surface area <109 g/m^2^ (women) and <132 g/m^2^ (men), regardless of the hypertension stage. Patients with hemodynamically significant valvular heart disease, a previous diagnosis of FD, previous history of any disease known to be associated with LVH, and familial history of autosomal dominant HCM or FD were also excluded.

Informed consent was obtained from all patients in accordance with the guidelines of the Ethics Committee (İstanbul University Cerrahpaşa Faculty of Medicine. Date: 4 December 2012 No: 83045809/3828). Evaluation of patients included medical history, clinical examination, 12-lead electrocardiography, and M-mode, two-dimensional (2-D), and Doppler echocardiography. Trans-thoracic echocardiography studies were performed using a Philips iE33 echocardiography machine and X5 transducer (Philips Healthcare, Andover, MA, USA) with the patient in the left lateral decubitus position. The standard evaluation included M-mode, 2-D, and Doppler studies according to the recommendations of the American Society of Echocardiography (14). LV ejection fraction was calculated from apical four-chamber views by manually tracing end-diastolic and end-systolic endocardial borders, using Simpson’s method. The maximum LV wall thickness was defined as the greatest end-diastolic thickness in any myocardial segment. The LV end-diastolic diameter and end-systolic diameter were measured from M-mode and 2-D images obtained from parasternal long-axis views, and the LV mass index was calculated using Devereux’s formula [0.80 × (1.04 (ST + PWT + LVID)^3^ − LVID^3^ + 0.6 gr]. When the diagnosis of HCM was established, patients were examined for the presence of LV outflow tract obstruction with Doppler echocardiography. The widely accepted cut-off value for instantaneous peak LV outflow tract pressure gradient is ≥30 mm Hg at rest, or during physiological provocation such as the Valsalva maneuver, standing, and exercise (2). Blood analyses were only performed in patients who had LVH without LV outflow tract obstruction at rest, or after provocation, as required by the protocol.

The presence of biochemical and genetic markers of FD was assessed using measurements of plasma AGE activity and genetic analysis as appropriate. Peripheral venous blood samples were collected in EDTA tubes (2 mL) from each patient and sent in a blinded fashion to an external laboratory that specialized in the diagnosis of genetic diseases. GLA gene sequence analyses were performed in all patients, whereas levels of plasma AGE activity were measured in patients with mutations in the GLA gene.

### Mutation analysis - polymerase chain reaction - sequencing

The seven exons of the GLA gene were amplified by polymerase chain reaction with specific primers and sequenced by the Sanger method on a Genetic Analyzer (Applied Biosystems Inc.). Results were analyzed using the software SeqScape 2.5.0 (Applied Biosystems Inc.). DNA was extracted with a QIAamp DNA Blood Mini Kit (Qiagen Inc.). Seven pairs of polymerase chain reaction primers were designed to amplify the seven exons encoding the GLA gene. The polymerase chain reaction amplifications were carried out using Taq DNA polymerase (PhireII HS, Thermo Inc.) and a polymerase chain reaction protocol with an initial hold of 1 minutes at 95 °C, 45 cycles (of 10 seconds at 95 °C, 10 seconds at 60 °C, and 20 seconds at 72 °C), and then a final extension of 1 minute at 72 °C. After the thermal cycle protocol for polymerase chain reaction, the product was checked using 2% agarose gel electrophoresis. Polymerase chain reaction products were purified using the ZR-96 DNA Sequencing Clean-up Kit (Zymo Research Corp.), and the purified products were sequenced bidirectionally on an ABI 3130 capillary gel electrophoresis system (Applied Biosystems Inc.) according to the manufacturer’s protocol. The exons of the gene and the exon-intron connections were analyzed by SeqScape 2.5.0 (Applied Biosystems Inc.) software, and the sequence variations were determined.

### Measurement of α-gal A - enzyme activity

Determination of AGE activity was based on a dried blood spot test that was used to perform the AGE activity study by a fluorimetric method. The normal range of α-gal A activity was defined as ≥3.3 μmol/L/hour.

### Statistical analysis

Demographic characteristics and echocardiographic parameters of all screened patients were analyzed. Data were submitted to descriptive analysis using the Statistical Package for the Social Sciences program, version 19.0 (SPSS Statistics IBM^®^), and were expressed as numbers or percentages or mean values ± standard deviation.

## RESULTS

We studied 80 patients with a clinical diagnosis of non-obstructive HCM. Table 1 shows the clinical characteristics of the patients with HCM in the present study. Patients’ ages ranged from 18 to 65 years (mean age, 41.5±12.7 years).

### Patients with FD

We found a mutation of the GLA gene in two patients (2.50%) and a clinical diagnosis of FD with cardiac involvement was finally made. The clinical characteristics of these two patients and the results of genetic analysis are shown in Table 2. After the initial diagnosis of FD, all patients were referred to a Metabolic Disease specialist for follow-up, treatment, and genetic counseling.

**Patient 1: **A 49-year-old male patient was referred to the cardiology outpatient clinic for echocardiography. The patient had no symptoms until the age of 30 years, the time of onset of fatigue, weakness, and palpitation. Functional capacity was New York Heart Association (NYHA) II at the time of evaluation. Systemic arterial pressure was 134/82 mm Hg, heart rate was rhythmic, and 84 beats per minute. Electrocardiography and echocardiography showed LVH. A normal LV ejection fraction (60%) was noted along with concentric LVH. He had an abnormally low AGE activity (0.5 μmol/L/hour), and the lysoGb3 rate was high (7.8 ng/mL). The results of the molecular analysis for FD indicated the hemizygous mutation NM_000169.2:c.334C>T(p.Arg112Cys) (rs104894834) (Figure 2). This mutation was identified according to ACMG-2015 criteria as “likely pathogenic”. After the establishment of the FD diagnosis, a further clinical assessment was carried out. He had no angiokeratomas, and his eye examination was normal. His blood biochemical evaluation showed elevated levels of urea and creatinine (end-stage renal disease). His urinalysis detected proteinuria.

**Patient 2:** A 38-year-old previously healthy male patient was hospitalized because of progressive dyspnea, darkening of the skin, abdominal pain, and extensive swelling all over the body, which were evident for one year. His blood pressure was 170/100 mm Hg, and his pulse was rhythmic at 80 beats per minute. He was referred to the echocardiography laboratory due to the presence of hypertension and New York Heart Association-III dyspnea on exertion. Electrocardiography and echocardiography showed LVH. Concentric hypertrophy that especially involved the mid and apical portions of the left ventricle, as well as the right ventricular free wall was detected. Also, there was a thickening of the mitral and aortic valves resulting in mild valvular regurgitation. A mild global LV hypokinesia with a LV ejection fraction of 44% was noted. Physical examination revealed bilateral crackles in the basal and mid lung fields. Bilateral pitting pretibial edema was detected. Standard blood biochemistry analysis was unremarkable. His urinalysis detected proteinuria. He had an abnormally low AGE activity (0.4 μmol/L/hour), and the lysoGb3 rate was high (5.1 ng/mL). Genetic analysis detected the hemizygous NM_000169.2:c.902G>A(p.Arg301Gln) (rs104894828) mutation diagnostic of FD (Figure 3). This mutation was identified according to ACMG-2015 criteria as being “likely pathogenic”.

## DISCUSSION

FD is a lysosomal storage disease characterized by deficient activity or absence of AGE, leading to systemic, primary lysosomal accumulation of globotriaosylceramide. The accumulation process is usually slow, and life-threatening complications often occur in adulthood, including renal failure, cardiovascular dysfunction, neuropathy, and stroke (4).

In our study, 80 patients, who were referred to our clinic for various clinical indications and in whom unexplained LVH were found were selected for gene mutations and plasma enzyme analysis. FD was detected in two patients (2.50%). In our study, all patients with FD were male. Screening for FD is usually based on the activity of AGE in plasma, especially for male patients who usually present with low or absent enzymatic activity. Female patients frequently have AGE activity that falls within the normal range owing to random X-chromosomal inactivation and usually requires gene sequence analysis for FD diagnosis. Gene sequence analysis (all coding regions and exon-intron ports) and GLA gene mutation are a definitive diagnosis of FD (15). In order to maximize the chances of detecting actual index FD cases among patients with unexplained LVH and no prior diagnosis of FD, only patients with non-obstructive HCM were selected to be screened for the presence of FD with biochemical and genetic analyses. This approach was as also chosen in the study protocol to establish a more cost-effective way of diagnosing FD while making better use of the limited available funds.

Cardiovascular involvement is the most important cause of mortality in patients with FD (16). Cardiac involvement is observed in 60% of patients at the time of diagnosis. In patients with FD and without treatment, LV mass and wall thickness increase with age (5). In our study, we detected concentric LVH in two patients. The application of newer echocardiographic techniques could have allowed the detection of FD patients with milder forms of cardiac involvement. On the other hand, we suggest that relying on conventional echocardiographic parameters seems to be a better way to incorporate a screening study into the daily practice of busy echocardiography laboratories.

**Figure 1:**
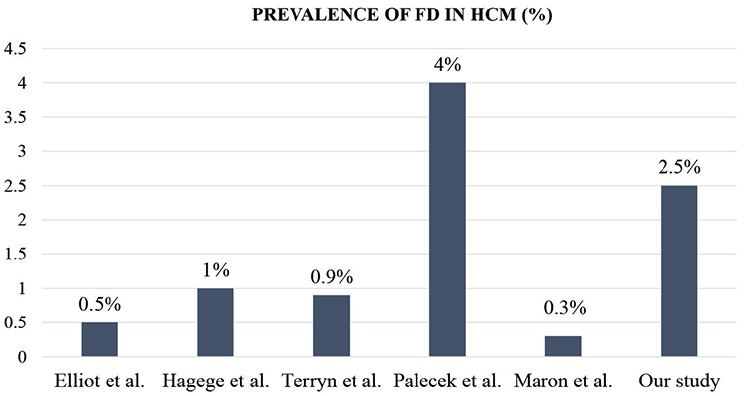
Prevelence of Fabry disease in various populations with hypertrophic cardiomyopathy patients. FD: Fabry disease; HCM: hypertrophic cardiomyopathy

**Figure 2:**
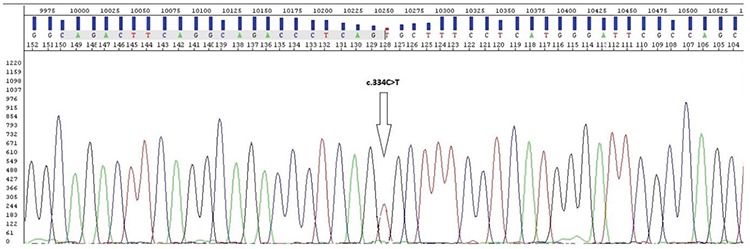
In genetic analysis c.334C>T(p.Arg112Cys) mutation diagnostic of Fabry disease was detected.

**Figure 3:**
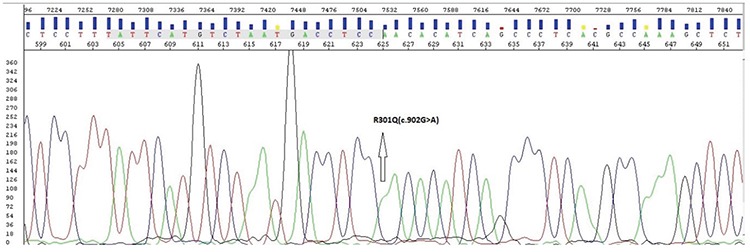
In genetic analysis c.902G>A(p.Arg301Gln) mutation diagnostic of Fabry disease was detected.

The exact incidence and prevalence of FD are unknown because of atypical or oligosymptomatic forms. Actual incidence and prevalence may be higher than expected (17). In previous studies of FD in patients with unexplained LVH, the prevalence of FD varied and ranged from 1% to 12% (Figure 1) (7,8,9,10,11,12,13). The differences between the populations screened and the screening methods used to measure AGE activity may explain the differences in prevalence reported by these studies. Elliot et al. (7) identified seven patients with FD in a European population cohort of 1386 patients (0.5%) with HCM or unexplained LVH. Hagege et al. (8) performed systematic screening for FD in the French population in patients with a diagnosis of HCM by an α-gal A assay on dried blood spots using a filter paper test, and they identified four patients with FD out of 278 men (1%). Terryn et al. (9) found five patients with FD in a Belgian cohort of 560 patients (0.9%) with unexplained LVH. Palecek et al. (10) identified four patients with FD in the Czech population among a cohort of 100 patients (4%) with LVH. Vieitez et al. (11) found that 21 patients in the Spanish population with FD among 805 patients (2.6%) with clinical symptoms or signs associated with FD. Maron et al. (12) performed screening in the North American population for FD in patients with a diagnosis of HCM, and they identified two patients with FD out of 585 patients (0.3%). In contrast, in 100 patients with HCM who underwent septal ablation, Ommen et al. (13) could not find any patients with FD. The reason for this negative finding could be that the hypertrophy secondary to FD is usually concentric and not associated with the generation of dynamic subaortic obstruction.

FD is a multi-systemic disease. The cardiologist has an essential role in screening and distinguishing LVH via ECG, echocardiography, or magnetic resonance imaging. Because FD is an X-linked disorder, identification of index FD cases raises important family screening considerations to identify relatives at risk for FD who may also be candidates for clinical monitoring and/or therapeutic intervention with enzyme replacement therapy (18,19).

The guidelines on HCM of the European Society of Cardiology have supported their message regarding the significance of investigating different reasons for LVH, for example, FD (2). However, screening for FD in our country remains suboptimal. A countrywide approach to identify FD among patients with non-obstructive HCM seems warranted.

## Figures and Tables

**Table 1:**
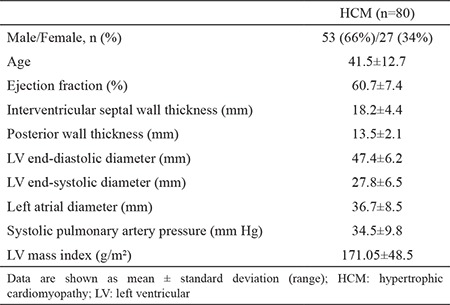
Clinical characteristics of study patients

**Table 2:**
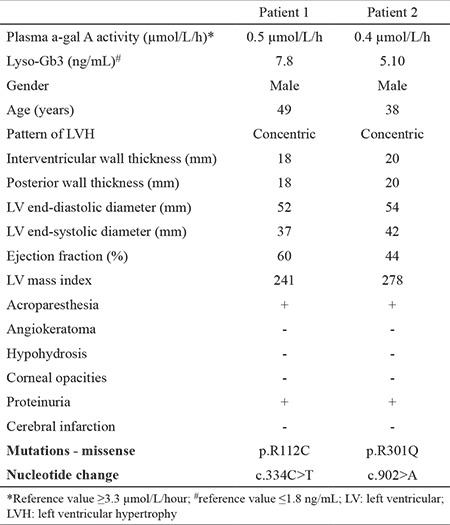
Clinical characteristics and genetic results in patients with Fabry disease
